# Cloning and Characterization of Two Putative P-Type ATPases from the Marine Microalga *Dunaliella maritima* Similar to Plant H^+^-ATPases and Their Gene Expression Analysis under Conditions of Hyperosmotic Salt Shock

**DOI:** 10.3390/plants10122667

**Published:** 2021-12-03

**Authors:** Dmitrii A. Matalin, Dmitrii E. Khramov, Alexey V. Shuvalov, Vadim S. Volkov, Yurii V. Balnokin, Larissa G. Popova

**Affiliations:** 1 K.A.Timiryazev Institute of Plant Physiology RAS, 127276 Moscow, Russia; dmatalin@mail.ru (D.A.M.); khramov.de@yandex.ru (D.E.K.); balnokin@mail.ru (Y.V.B.); 2 Engelhardt Institute of Molecular Biology RAS, 119991 Moscow, Russia; laursen1243@mail.ru

**Keywords:** *Dunaliella*, cloning, expression, H^+^-ATPase, microalgae, Na^+^-ATPase, qRT-PCR, salt shock, salt tolerance

## Abstract

The green microalga genus *Dunaliella* is mostly comprised of species that exhibit a wide range of salinity tolerance, including inhabitants of hyperhaline reservoirs. Na^+^ content in *Dunaliella* cells inhabiting saline environments is maintained at a fairly low level, comparable to that in the cells of freshwater organisms. However, despite a long history of studying the physiological and molecular mechanisms that ensure the ability of halotolerant *Dunaliella* species to survive at high concentrations of NaCl, the question of how *Dunaliella* cells remove excess Na^+^ ions entering from the environment is still debatable. For thermodynamic reasons it should be a primary active mechanism; for example, via a Na^+^-transporting ATPase, but the molecular identification of Na^+^-transporting mechanism in *Dunaliella* has not yet been carried out. Formerly, in the euryhaline alga *D. maritima*, we functionally identified Na^+^-transporting P-type ATPase in experiments with plasma membrane (PM) vesicles which were isolated from this alga. Here we describe the cloning of two putative P-type ATPases from *D. maritima*, *Dm*HA1 and *Dm*HA2. Phylogenetic analysis showed that both ATPases belong to the clade of proton P-type ATPases, but the similarity between *Dm*HA1 and *Dm*HA2 is not high. The expression of *Dm*HA1 and *Dm*HA2 in *D. maritima* cells under hyperosmotic salt shock was studied by qRT-PCR. Expression of *DmHA1* gene decreases and remains at a relatively low level during the response of *D. maritima* cells to hyperosmotic salt shock. In contrast, expression of *DmHA2* increases under hyperosmotic salt shock. This indicates that *Dm*HA2 is important for overcoming hyperosmotic salt stress by the algal cells and as an ATPase it is likely directly involved in transport of Na^+^ ions. We assume that it is the *Dm*HA2 ATPase that represents the Na^+^-transporting ATPase.

## 1. Introduction

The genus of motile green unicellular algae *Dunaliella* belongs to the family Dunaliellaceae, which was described more than a hundred years ago [1]. Since then, representatives of this genus have become convenient model organisms in plant cell physiology for studying the cellular mechanisms of adaptation to unfavorable environmental factors, particularly, to increased salinity [2,3]. The genus *Dunaliella* includes freshwater species (*D. acodophila*, *D. lateralis*, *D. flagellate*), the marine/oligohaline/euryhaline species with optimum salinity for growth of about 2 to 4% NaCl (*D. tertiolecta, D. polymorpha, D. maritima*) and hypersaline/halophilic species with optimum salinity for growth >6% NaCl (*D. parva, D. salina, D. bioculata*) [4]. Primarily, representatives of the genus *Dunaliella* are known as inhabitants of hypersaline reservoirs [2,5]. For example, *D. salina* and *D. parva*, which are found in saturated brines where the NaCl concentration reaches 100‰ (app. 1.7 M NaCl), provide the major part of biomass production of saline lakes [1]. Some *Dunaliella* species (*D. tertiolecta, D. maritima*) are marine/euhaline organisms; they show moderate resistance to NaCl compared to *D. salina* and *D. parva*. The optimal growth of these species is observed at 28–32% (app. 0.5 M NaCl), but these species are also able to grow at very high concentrations of NaCl reaching 1.5 M [6,7].

The ability of *Dunaliella* to grow in water reservoirs with variable salt concentration, is associated with the absence of a rigid cell wall and suggests that *Dunaliella* has effective systems of ionic and osmotic regulation necessary for life under changing environmental conditions. The question of maintaining osmotic balance in *Dunaliella* cells under conditions of high salinity has been well investigated in numerous studies. It was shown that the osmotic balance in *Dunaliella* is supported by the synthesis of a compatible osmolyte glycerol, which, depending on the concentration of NaCl in the medium, can accumulate to reach intracellular concentrations of 7.8 M [8,9,10,11].

At high concentrations Na^+^ is toxic to cellular metabolism. High Na^+^ concentrations over 100 mM have an inhibitory effect on protein synthesis, both in glycophytes and halophytes, including *Dunaliella* species [10,12,13,14,15,16,17]. Sodium is competing with potassium for allosteric sites of enzymes which leads to violations of cellular functions [18]. Sodium is also interacting with ion channels. For example, Na^+^ ions change the gating of potassium outward rectifying currents (most likely carried by Shaker type K^+^ channels) in root protoplasts of halophyte plant *Thellungiella* [19]. Moreover at the cellular level salt stress induces apoptosis [20] (briefly reviewed in [21,22]).

Like in all living organisms, Na^+^ content in *Dunaliella* cells inhabiting saline environments is maintained at a fairly low level. Both in *Dunaliella* growing at 0.5 M NaCl and in species growing at 4 M NaCl in the medium, intracellular concentrations of Na^+^ do not exceed 100 mM [23,24,25,26,27,28]. An interesting example is the halotolerant alga *D. salina*, which is a good unicellular eukaryotic model for studying salinity tolerance within the range of 0.05–5.5 M NaCl [25]. The cells of *D. salina* are small with a length about 10–11 μm, width of 6 μm and volume around 200 fL (or even smaller dimensions with a volume around 90–100 fL) [4]. Cytoplasmic Na^+^ concentrations of about 90 mM (88 ± 28 mM) were reported in the alga using ^23^Na-NMR spectroscopy [24] and were nearly the same (within the error of measurements) in the algal cells adapted to a wide range of external Na^+^, from 0.1 to 4 M. Similar or even lower sodium concentrations below 100 mM were measured by other methods for the alga under 0.5–4 M sodium treatment [25,27].

However, despite a long history of studying the physiological and molecular mechanisms that ensure the ability of halotolerant *Dunaliella* species to survive at high concentrations of NaCl in the environment, the question of how *Dunaliella* cells get rid of excess Na^+^ ions entering from the external environment is still debatable. In higher plants—organisms of the plant kingdom, which also include Dunaliellaceae—Na^+^ homeostasis in cells is maintained by means of ΔμH^+^-dependent Na^+^/H^+^ antiporters at the plasma membrane and tonoplast, which export Na^+^ from the cytoplasm to the external environment or to vacuoles (reviewed in [29,30,31]). The functioning of Na^+^/H^+^ antiporters is energized by active H^+^-pumps that, at the expenses of ATP, produce H^+^ gradients at the cell membranes. These H^+^-pumps are P-type H^+^-ATPase resident in plasma membranes and V-type H^+^-ATPase and H^+^-translocating pyrophosphatase of tonoplast [32]. Plasma membrane H^+^-ATPases form a highly conservative subgroup of P-type ATPases. H^+^-ATPases are ubiquitous and essential enzymes in yeast and higher plants where they ensure the driving force for uptake of nutrients, cytoplasmic pH-stat and regulation of cellular volume (reviewed in [33,34,35]). P-type H^+^-ATPases have also been found in algae of various taxa [35,36,37,38,39,40].

It is important to mention one more structural peculiarity of Dunaliellaceae: along with the absence of a rigid cell wall, they also lack a large central vacuole. Since the alga has no central vacuole, the plasma membrane ATPase is considered to be the major means by which the intracellular Na^+^ concentration is regulated. However, calculations demonstrated that under conditions which are typical for the habitats of halotolerant microalgae —i.e., in environments where high concentrations of NaCl are combined with alkaline pH —there are thermodynamic restrictions for the export of Na^+^ from cells of these organisms by means of ΔµH^+^-dependent Na^+^/H^+^ antiporter of the plasma membrane energized by H^+^-ATPase [41]. If export of Na^+^ from cytoplasm to the external medium by a secondary active ΔµH^+^-dependent Na^+^/H^+^ antiporter is not possible, then the Na^+^ homeostasis of the cytoplasm has to be provided by a primary active mechanism, for example, by a Na^+^-transporting ATPase, which will directly export Na^+^ from the cytoplasm at the expenses of energy of ATP hydrolysis. The examples of such Na^+^-pumps in eukaryotic kingdoms are the well-characterized Na^+^, K^+^-ATPase of animal cells [42] (reviewed in [43,44,45]) and the yeast-type Na^+^-ATPase, ENA ATPase, which is found in different yeast species [46,47]. These enzymes, like the proton ATPases of the plant cell plasmalemma, belong to the family of P-type ATPases. P-type ATPases are relatively simply arranged integral membrane proteins that couple hydrolysis of ATP with transfer of small cations (Na^+^, K^+^, Ca^2+^, H^+^), heavy metal ions and phospholipids through biological membranes against their electrochemical gradients (reviewed in [48,49]). A characteristic feature of P-type ATPases is the formation of a transient phosphorylated intermediate during the catalytic cycle [50].

In algae of genus *Dunaliella*, a Na^+^-transporting P-type ATPase was functionally identified in experiments with plasma membrane (PM) vesicles, which were isolated from the cells of green euryhaline microalga *D. maritima*. Under conditions when there was no proton gradient at the vesicle membrane ATP-dependent uptake of ^22^Na^+^ by PM vesicles occurred thus indicating the functioning of a primary-energized Na^+^-transporting mechanism in *D. maritima* PM, namely, a Na^+^-transporting ATPase [51]. This enzyme as the other eukaryotic Na^+^-ATPases is a P-type ATPase, since it has a characteristic feature of these ATPases, namely, sensitivity to micromolar concentrations of orthovanadate. As with the Na^+^-ATPase of microalga *Tetraselmis viridis*, the Na^+^-ATPase of *D. maritima* differs from both Na^+^, K^+^-ATPase of animal cells and yeast ENA ATPases based on a number of principal functional characteristics. The Na^+^-ATPase of *D. maritima* does not transport K^+^, it is an electrogenic uniporter [52]. This uniporter feature discriminates the Na^+^-ATPase of *D. maritima* from the Na^+^-ATPase of *T. viridis*, which uses Na^+^ and H^+^ as counterions. However, similar to the Na^+^-ATPase of *T. viridis*, the Na^+^-ATPase of *D. maritima* is highly selective for Na^+^ [51,52]. Nevertheless, even though the Na^+^-ATPase of *D. maritima* was discovered at the functional level over 20 years ago, the molecular identification of this enzyme has not yet been carried out.

Experiments to clone P-type ATPases from different *Dunaliella* species have been undertaken [53,54,55]. The cloned ATPases were either structurally similar to H^+^-ATPases of plants (ATPases from *D. bioculata* [53], *D. acidophyla* and *D. salina* [54]) or similar to Ca^2+^-ATPases (ATPase from *D. bioculata* [55]), but an enzyme which could be classified as a subgroup of Na^+^-transporting ATPases by its general structural characteristics was not found.

Ambiguity over the molecular identification of the mechanism responsible for the export of Na^+^ from *Dunaliella* cells is increased by the results of our bioinformatic study. In order to identify the gene of Na^+^-ATPase for algae belonging to the genus *Dunaliella* we assembled *de novo* several transcriptomes of the microalga *D. tertiolecta* based on individual libraries of short RNA reads from *D. tertiolecta* available in free access in the Sequence Read Archive (SRA, NCBI) database. The assembled transcriptomes were examined *in silico* for possible signatures for P-type ATPases [56]. Coding sequences (CDS) for various P-type ATPases were found, but contrary to expectations, none of the assembled *D. tertiolecta* transcriptomes demonstrated a nucleotide sequence encoding a protein that could be unambiguously assigned to the Na^+^-ATPase group. However, contigs containing CDS for two different hypothetical H^+^-ATPases, HA1 (molecular weight 1131 aa) and HA2 (molecular weight 923 aa) have been identified. Contigs containing full-length CDS for these ATPases were found in all assembled transcriptomes indicating their relatively high abundance in the total transcript pool. The function of the proteins was supposed by their location on a P-type ATPase cladogram in the same clade with H^+^-ATPases.

The similarity between ATPases HA1 (termed here as *Dt*HA1) and HA2 (termed as *Dt*HA2) was not high. ATPase *Dt*HA1 was identical (100% identical to amino acid residues) to H^+^-ATPase of the plasma membrane of *D. bioculata* (GenBank CAA52107.1) and showed a high degree of similarity with H^+^-ATPase from the acidophilic alga *D. acidophila* (P54210.1, UniProtKB/Swiss Prot Database). The other putative *D. tertiolecta* ATPase, *Dt*HA2, was similar (99% identical amino acid residues) to P-type ATPase of the halotolerant microalga *D. salina* (ABB88698.1, GenBank database). The phylogenetic analysis demonstrated that ATPase *Dt*HA1 is homologous to the proton pumps of higher plants, while ATPase *Dt*HA2 is homologous to H^+^-ATPases of microalgae and parasitic protists [56].

Based on the assumption that the two different proton pumps in the plasmalemma of *D. tertiolecta* were unlikely to have a similar function, it was hypothesized that one of the predicted enzymes (transcripts of which were identified in *D. tertiolecta* transcriptome), *Dt*HA1, which is similar to the well-characterized H^+^-ATPases of higher plants and the acidophilic microalga *D. acidophila* [54], is an H^+^-ATPase and transfers protons while another ATPase, *Dt*HA2, may carry Na^+^ ions.

This hypothesis is supported by some data obtained in the present work. Based on the sequences encoding *Dt*HA1 and *Dt*HA2 ATPases in the *de novo* assembled *D. tertiolecta* transcriptomes, primers were designed to amplify the coding sequences of homologous ATPases, *Dm*HA1 and *Dm*HA2 from the green euryhaline microalga *D. maritima*, which is closely related to alga *D. tertiolecta* [4,57] and was extensively characterized in our laboratory [7,14,51,52,58,59]. As mentioned above, the existence of Na^+^-transporting ATPase in the plasma membrane of *D. maritima* was shown at the functional level [51]. The sequences *DmHA1* and *DmHA*2 were cloned, their expression was studied in *D. maritima* cells under hyperosmotic salt shock, the similarities and differences in the structures of proteins *Dm*HA1 and *Dm*HA2 were analyzed *in silico*. The data obtained indicate that ATPase *Dm*HA2 may participate in export of Na^+^ ions from *D. maritima* cells.

## 2. Materials and Methods

### 2.1. The Object of the Study and Conditions of Cultivation

The object of the study was the green euryhaline microalga *Dunaliella maritima* [60]. A suspension culture of the alga was grown in a liquid medium containing 0.5 M NaCl, pH 8: the complete composition of the medium is close to that of seawater and is given in [41]. *D. maritima* culture was cultured in glass vessels with a volume of 1 L, constantly bubbled with air containing 1.5% CO_2_, and illuminated with white light from LB-20 fluorescent lamps for 14 h a day. Irradiance was 225 μmol photons m^−2^ s^−1^.

A number of experiments also used a *D. maritima* culture growing in a medium with a low concentration of NaCl (0.1 M NaCl; “low-salt” culture). “Low-salt” culture was obtained by acclimating a “high-salt” culture of alga growing at 0.5 M NaCl to 0.1 M NaCl in the medium for at least 2 months. Passages of the algal culture to a fresh nutrient medium were carried out weekly.

### 2.2. Isolation of Total RNA from D. maritima Cells

For isolation of total RNA from *D. maritima* cells, 200 mL of alga cell suspension at the late logarithmic growth stage were taken, the culture density was about 1.5 × 10^7^ cells/mL. The total RNA was isolated by the hot phenolic method according to de Vries et al. [61]. To remove the residual contaminant of genomic DNA, total RNA samples were treated with DNase I (“Fermentas”, Thermo Fisher Scientific, Inc., Waltham, MA, USA) according to the manufacturer’s protocol. RNA concentration and quality assessment by the ratio A_260_/A_280_ were measured by NanoDrop ND1000 (Thermo Fisher Scientific, Inc., Waltham, MA, USA). The quality of isolated RNA was also confirmed by electrophoresis in 1% agarose gel.

### 2.3. cDNA Synthesis on the Total RNA Template

Samples of total RNA isolated from *D. maritima* cells were used to synthesize total cDNA in a reverse transcription reaction with MMLV-revertase (“Evrogen”, Moscow, Russia) and a 12-dTVN oligo-dT primer. The reaction was carried out according to the manufacturer’s protocol.

### 2.4. Amplification of the DmHA2 Partial Coding Sequence

The partial coding fragment of ATPase *Dm*HA2 was amplified on the template of the obtained total cDNA using degenerate primers (F: 5′-gAYAARACYggCACYCTCAC-3 ‘and R: 5′-TCRTTCACRCCATCACCYgT-3′) and the Encyclo Plus PCR kit (“Evrogen”, Moscow, Russia).

### 2.5. Amplification of the Full-Length Sequences Encoding the DmHA1 and DmHA2

Amplification of the full-length *Dm*HA1 and *Dm*HA2 cDNA sequences was performed on the template of the total cDNA obtained using CloneAmp^TM^ HiFi PCR Premix kit optimized for high-fidelity PCR (“TaKaRa”/Takara Bio Inc., Shiga, Japan; cat # 638916). For amplification, gene-specific primers (Supplementary Table S1) were used, selected based on the assumed homology between the nucleotide sequences coding *D. maritima* ATPases and the corresponding sequences found in the assembled transcriptome of a closely related alga *D. tertiolecta* [56] (Supplementary S2). The primers were designed using the SnapGene Viewer software (from Insightful Science; available at https://www.snapgene.com/, accessed on 1 December 2021).

Additionally, the adaptors of 15–17 bp were added to the 5′-ends of the primers to make them complementary to the ends of linearized vector pMB1. The procedure was necessary for the vector constructions. The first 20 cycles of amplification were done using polymerase Encyclo (“Evrogen”, Moscow, Russia) according to the protocol of the company, then 1 μL of the resulting PCR mix was used as a template for the further 32 cycles of amplification with the same primers and high fidelity polymerase from CloneAmp^TM^ HiFi PCR Premix kit (“TaKaRa”). Amplicons from the final PCR mix were used for assemblies of constructs based on vector pMB1, which was initially linearized by inverse PCR [62]. To linearize the vector, we also used CloneAmp^TM^ HiFi PCR Premix kit and the corresponding primers (Supplementary Table S1). Ligation of amplicons and the linearized pMB1 was performed using Gibson Assembly^®^ Cloning Kit (“New England Biolabs”, Ipswich, MA, USA) according to the protocol for the kit. The resulting constructs were routinely propagated in *E. coli* cells. The cloned *D. maritima* ATPase sequences were annotated in GenBank.

### 2.6. Analysis of DmHA1 and DmHA2 Expression under Hyperosmotic Salt Shock

Hyperosmotic salt shock for the cells of the alga *D. maritima* was created by adding 4.5 M NaCl solution to the “low-salt” cell suspension of the alga to a final concentration of 0.5 M NaCl. The cell suspension aliquots (200 mL) were taken at time intervals of 5, 15, 30, 60, 90, 120 and 180 min after the addition of NaCl and frozen in liquid nitrogen. Samples for cells growing at 0.1 M and at 0.5 M NaCl were also collected. Total RNA preparations were then obtained from the cell aliquots and used for cDNA synthesis in a reverse transcription reaction. The relative contents of *Dm*HA1 cDNA and *Dm*HA2 cDNA in the samples obtained were analyzed by quantitative real-time RT-PCR (qRT-PCR) with the LightCycler^®^ 96 Instrument (Roche Diagnostics Corporation, Indianopolis, IN, USA). Reaction mix (20 μL) included 5 μL (100 ng) of cDNA template, 4 μL of ready-made reaction mixture with intercalating fluorescent dye SYBR Green (5 × SYBR Green I qPCRmix-HS SYBR, “Evrogen”, Moscow, Russia), 1 μL of each primer for qRT-PCR (Table S1) (the final concentration of each primer was 0.5 μM), 9 μL mQH_2_O. The amplification program was the following: 5 min at 95 °C, 45 cycles of 20 s at 95 °C, 20 s at 58 °C, 20 s at 72 °C. The specificity of PCR products was confirmed by the melting curve at the end of the amplification cycle. The sizes of the target fragments were 186 bp for *Dm*HA1 and 196 bp for *Dm*HA2. Gene of *Dunaliella* β-tubulin was selected as a reference gene, it was cloned by us and annotated in GenBank (ID: MW679534). The size of the synthesized fragment of β-tubulin was 127 bp.

The data obtained were processed using the software for the LightCycler^®^ 96 Instrument (Roche Diagnostics Corporation, Indianopolis, IN, USA), where the 2^−ΔΔCt^ method is used for relative transcription level calculations. The fold changes are represented as (final values–initial values)/initial values. The results are for 3 biological replicates.

### 2.7. Determination of Na^+^ Content in D. maritima Cells under Hyperosmotic Salt Shock

Aliquots (10 mL) of alga cell suspension were taken before and at 5, 10, 15, 20, 30, 40, 60, 90, 120, 150 and 180 min after a sharp increase in the salt concentration in the medium. The alga cells were separated from the external medium by centrifugation through a layer of isotonic washing solution containing 1 M mannitol and 20 mM Ca(NO_3_)_2_ [7]. Distilled water (3 mL) was added to the cell precipitate, which caused cell lysis. Then the cell fragments were precipitated by centrifugation and the content of Na^+^ ions in the supernatant was determined using a flame photometer Leki FP 640 (“Leki”, Finland). To calculate intracellular ion concentrations, the quantity of ions obtained was attributed to the total volume of cells in the sample. The cell volume was determined by the Okamoto and Suzuki method [63] by the difference in the electrical conductivity of the medium and the cell suspension using the OK-102/1 conductometer (Radelkis, Budapest, Hungary).

### 2.8. Bioinformatic Methods

The frequency of the codons usage in the green microalgae for design of the degenerate primers was determined according to the Codon Usage Database website (http://www.kazusa.or.jp, accessed on 1 December 2021). Virtual translation of nucleotide sequences into amino acid sequences was carried out using the on-line service on the ExPASy portal (http://web.expasy.org/translate/, accessed on 1 December 2021). Molecular weight, theoretical isoelectric point and the grand average of hydropathicity (GRAVY) of the ATPase proteins were analyzed by the ExPASy compute MW/pI tool (http://web.expasy.org/protparam/, accessed on 1 December 2021). The subcellular localizations of the ATPases were predicted using the on-line tools WoLF PSORT II prediction on the GenScript server (https://www.genscript.com/tools/wolf-psort/, accessed on 1 December 2021). The homology of amino acid sequences of *Dm*HA1 and *Dm*HA2 was determined using Protein BLAST (Basic Local Alignment Search Tool) at NCBI portal (National Center Biotechnology Information, http://www.ncbi.nlm.nih.gov, accessed on 1 December 2021). To determine the phylogenetic relations of *Dm*HA1 and *Dm*HA2 with the known P-type H^+^- and Na^+^- ATPases, the amino acid sequences of the ATPases were extracted from NCBI portal. Multiple alignment of the ATPase sequences was performed using ClustalW analysis in “MEGA X” software [64]. The phylogenetic tree of the ATPases was constructed using the maximum likelihood method also by means of “MEGA X” software. The topology of *Dm*HA1 and *Dm*HA2 ATPases and the location of the transmembrane domains (TMD) were predicted using the CCTOP (Constrained Consensus TOPology) online service (http://cctop.enzim.ttk.mta.hu, accessed on 1 December 2021), which predicts a consistent model using 10 TMD prediction software tools (HMMTOP, Memsat, Octopus, Philius, Phobius, Pro, Prodiv, Scampi, ScampiMsa, TMHMM).

## 3. Results and Discussion

In the first stages of the study, cloning of a partial coding sequence (CDS) of the P-type ATPase of the microalgae *D. maritima* was carried out. This was done to check the similarity of the sequences encoding the *D. maritima* ATPases with the orthologous sequences from the phylogenetically related microalga *D. tertiolecta* [57]. The *D. tertiolecta* sequences were previously identified by us in the *de novo* assembled transcriptome of this microalga [56]. To amplify the fragment of CDS for P-type ATPase from *D. maritima* we used degenerate primers which were designed according to the structural features of P-type ATPases.

P-type ATPases have a relatively simple structure. Typically, these enzymes consist of a single catalytic subunit with a molecular weight of 90–140 kDa and have a similar three-dimensional organization though they may vary in size. These are integral membrane proteins containing, depending on the affiliation of the ATPase to a particular subfamily of P-type ATPases [65], 6–10 transmembrane segments (α-helices) which form a transmembrane domain, small and large cytoplasmic loops forming a large cytoplasmic domain and N- and C-ends lying in the cytoplasm [50]. However, despite the general similarity of tertiary structures, the primary structures of P-type ATPases generally are not very similar [65]. Nevertheless, in each enzyme belonging to this class, 8 regions in the amino acid sequence are present that have a high degree of homology among all P-type ATPases. In turn, there are highly conserved regions within these areas. As a rule, rather short amino acid motifs (PGD, PAD, TGES, DKTGTLT, KGAP, DPPR, MVTGD, TGDGVND) located in the catalytic cytoplasmic domain of the enzyme and involved in ATP binding and hydrolysis are strictly invariable [66,67].

The most conserved sequence present almost unchanged in all P-type ATPases, is the DKTGTLT sequence. It is located within a large cytoplasmic loop of the ATPase protein, its aspartate is phosphorylated during the catalytic cycle of the enzyme due to gamma phosphate transfer from ATP, and it is this autophosphorylation that is a characteristic feature of P-type ATPases. Another rather long conservative sequence typical for P-type ATPases, TGDGVND, is located in the “hinge” joint area. This sequence connects the large cytoplasmic domain with the C-terminal hydrophobic domain and is involved in conformational changes during the catalytic cycle of ATPase. The DKTGTLT and TGDGVND sequences are located at a distance of 300–400 amino acids from each other [67]. Degenerate primers for amplification of the fragment of P-type ATPase from *D. maritima* were designed based on these two highly conserved regions of amino acid sequences, taking into account the frequency of codon usage in green microalgae. With the degenerate primers, a 771 nucleotide cDNA fragment was amplified by RT-PCR on the total RNA template obtained from the cells of the microalga *D. maritima* growing at 0.5 M NaCl in the medium. The resulting fragment was sequenced (Supplementary S3). It turned out that it is completely identical to the part of the nucleotide sequence identified *in silico* in the *de novo* assembled transcriptome of *D. tertiolecta* and encoding an ATPase termed HA2 (*Dt*HA2) [56]. Consequently, we termed the corresponding sequence in *D. maritima DmHA2*.

The identity of nucleotide sequences encoding fragments of ATPases, *Dm*HA2 in *D. maritima* and *Dt*HA2 in *D. tertiolecta*, gave us reasons to use gene-specific primers (Supplementary Table S1) for amplification of full-size sequences encoding *Dm*HA1 and *Dm*HA2 of *D. maritima*. The design of the primers was carried out on the basis of full-size nucleotide sequences-orthologs, found *in silico* in *de novo* assembled transcriptome of the microalga *D. tertiolecta* (Supplementary S2). Using these primers, amplicons of 3631 and 2894 nucleotides in size were obtained on the template of the total RNA from *D. maritima* cells and annotated in GenBank (ID: MK510928.1 and KX832225.1, respectively). Significantly, these amplicons turned out to be almost identical to the contigs from the *de novo* assembled transcriptome of *D. tertiolecta* containing the coding sequences for *Dt*HA1 and *Dt*HA2 ATPases (Supplementary S2). The amplicons obtained contained full-length open reading frames for two proteins. Based on the deduced amino acid sequences these proteins were characterized as P-type ATPases and termed as *Dm*HA1 (1131 aa, GenBank with ID: QEH60479.1) and *Dm*HA2 (923 aa, GenBank ID: AQM50087.1). Remarkably, the amino acid sequences of *Dm*HA1 and *Dm*HA2 as well as their nucleotide sequences have a slight similarity: for paired alignment about 27% of identical and 42% of similar amino acid residues can be detected (Supplementary S4). Sequence analysis of *Dm*HA1 and *Dm*HA2 revealed that they possess the conserved motifs typical for P-type ATPases. Computed parameters of *Dm*HA1 and *Dm*HA2 are presented in Table 1.

**Figure 1:**
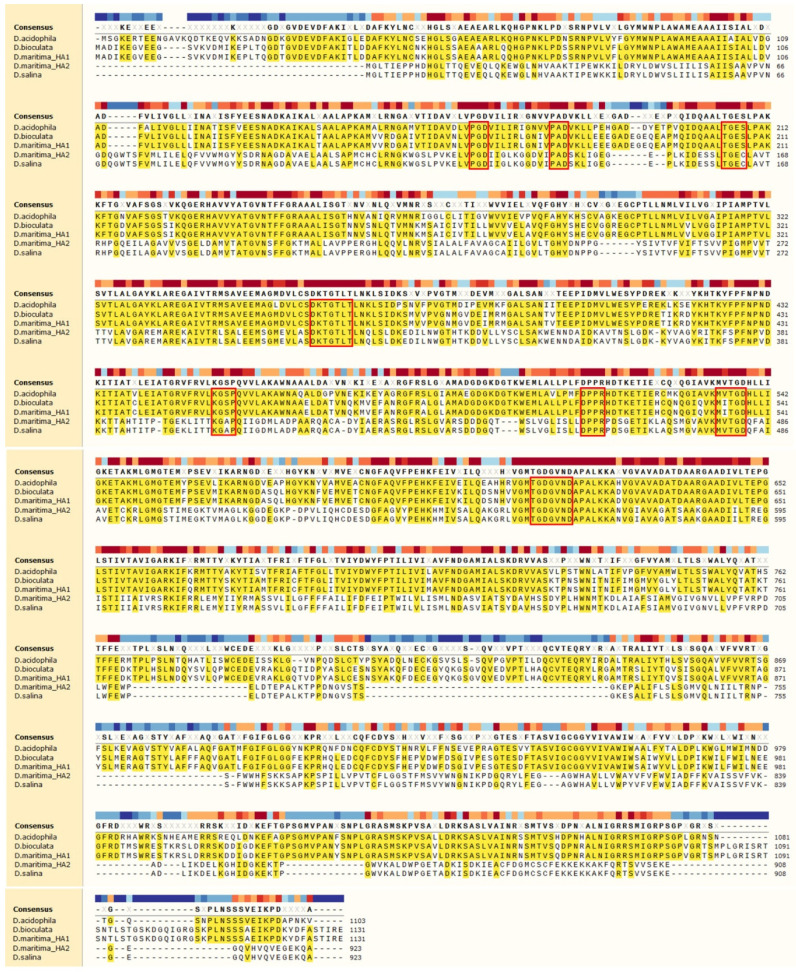
Multiple alignment of aa sequences of ATPases from *Dunaliella* species. The identical amino acids are shown in yellow background, and the conserved amino acids in P-type ATPases are highlighted by frames. The proteins for alignment: *D. acidophila*, P54210.1; *D. bioculata*, P54211.1; *D. maritima*_HA1, QEH60479.1; *D. maritima*_HA2, AQM50087.1; *D. salina*, ABB88698.1.

Cloned from *D. maritima* ATPases, *Dm*HA1 and *Dm*HA2, turned out to be very similar to cloned ATPases from other *Dunaliella* species (Figure 1, Table 1). For example, *Dm*HA1 is almost identical to H^+^-ATPase from *D. bioculata* (GenBank ID: P54211.1; 100% identical amino acids) and is very similar to H^+^-ATPase from *D. acidophila* (GenBank ID: P54210.1, about 75% of identical amino acids). The *Dm*HA2 ATPase is similar to the enzyme from the extremely halotolerant *D. salina* (GenBank ID: ABB88698.1, about 99% of identical amino acids).

**Figure 2:**
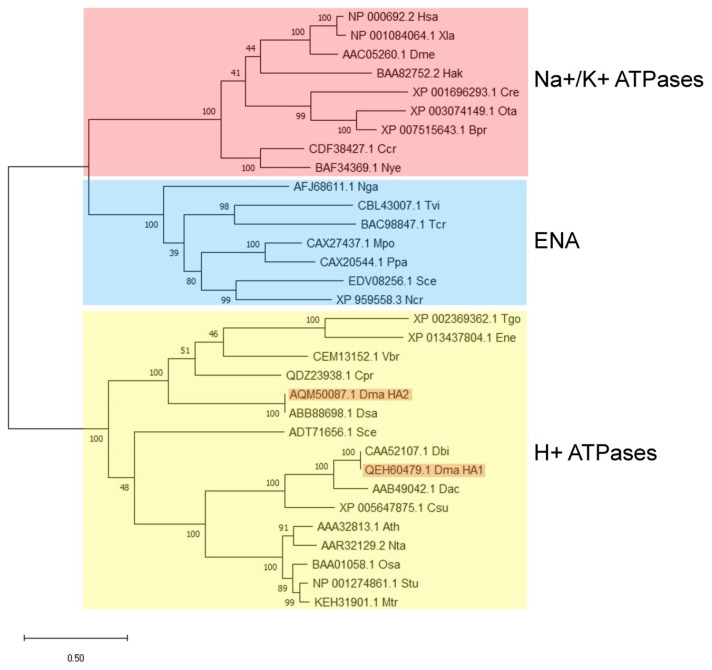
Cladogram of P-type H^+^-ATPases and Na^+^-ATPases from different organisms. Amino acid sequences from the following organisms are included in the analysis: Ath, *Arabidopsis thaliana*; Bpr, *Bathycoccus prasinos*; Ccr, *Chondrus crispus*; Cpr, *Chloropicon primus*; Cre, *Chlamidomonas reinhadtii*; Csu, *Coccomyxa subellipsoidea*; Dac, *Dunaliella acidophila*; Dbi, *Dunaliella bioculata;* Dma, *Dunaliella maritima*; Dme, *Drosophila melanogaster*; Dsa, *Dunaliella salina*; Ene, *Eimeria necatrix*; Hak, *Heterosigma akashiwo*; Has, *Homo sapiens*; Mpo, *Marchantia polymorpha*; Mtr, *Medicago truncatula*; Ncr, *Neurospora crassa*; Nga, *Nannochloropsis gaditana*; Nta, *Nicotiana tabacum*; Nye, *Neopyropia yezoensis;* Osa, *Oryza sativa*; Ota, *Ostreococcus tauri*; Ppa, *Physcomitrella patens*; Sce, *Saccharomyces cerevisiae*; Stu, *Solanum tuberosum*; Tcr, *Trypanosoma cruzi*; Tgo, *Toxoplasma gondii*; Tvi, *Tetraselmis viridis*; *Vbr, Vitrella brassicaformis*; Xla, *Xenopus laevis*.

Phylogenetic analysis showed that ATPases *Dm*HA1 and *Dm*HA2 belong to the clade of proton P-type ATPases (Figure 2). However, within the clade of putative proton ATPases, the two cloned *D. maritima* ATPases are located in different subclades: *Dm*HA1 protein is similar to the well-characterized proton pumps of higher plants and *S. cerevisiae*, while *Dm*HA2 ATPase is similar to the less studied putative proton ATPases of microalgae and parasitic protists. The transport of monovalent cations (as well as H^+^ transport) has not been demonstrated for the ATPases from this subclade. It should be noted that for most cases the coding sequences of proteins included in the second clade have not been determined experimentally but based on genomic sequencing while the functions of these proteins are predicted and have not been experimentally determined so far.

**Figure 3:**
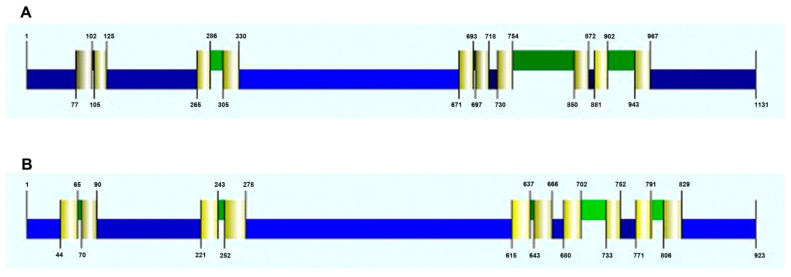
Predicted topology of *Dm*HA1 ((**A**), 1131 aa) and *Dm*HA2 ATPases ((**B**), 923 aa) and the location of the transmembrane domains (TMD) (colored pale yellow). Cytoplasmic areas (N-terminus, small and large cytoplasmic loops, C-terminus) are colored blue, extracellular protein areas are colored green. The figure illustrating the consistent model derived by the CCTOP (Constrained Consensus TOPology) online service (http://cctop.enzim.ttk.mta.hu, accessed on 1 December 2021) is taken from the Philius prediction (http://cctop.enzim.ttk.mta.hu, accessed on 1 December 2021).

Using programs for prediction of transmembrane domains in proteins we obtained consistent topological models for *Dm*HA1 and *Dm*HA2 (Figure 3). The modeling demonstrated that both are integral membrane proteins with 10 transmembrane segments. This number of transmembrane segments is typical for P-type ATPases which belong to subfamilies P2 (includes Na^+^-ATPases) and P3 (includes H^+^-ATPases) of P-type ATPases [67]. The analysis of structure for the two ATPases demonstrates that the highest similarity lies within the region of 100–700 amino acids (Supplementary S4). This region includes the small cytoplasmic domain, the 3rd and the 4th transmembrane helices and the large cytoplasmic domain, and it is the region which contains all the important conservative parts of P-type ATPases [66]. This region is linked to the details of the catalytic cycle that are common for all P-type ATPases independently of their ion selectivity: binding of ATP and Mg^2+^ ions as reaction cofactor, formation of phosphorylated intermediate, conformational changes of the enzyme [45]. The similarity of *Dm*HA1 and *Dm*HA2 within the hydrophobic C-terminal part of the proteins, where modeling predicted transmembrane segments M4, M5, M6 and M8 involved in the formation of the transmembrane pathway for the transferred cation and playing a critical role in its high-affinity binding [33], is not high. It may suggest that *Dm*HA1 and *Dm*HA2 transport different cations. It is also worth mentioning that sometimes the substitution of a single amino acid can change the ion selectivity of enzyme, in particular swopping H^+^ transport for that of Na^+^ ions [68].

Since the similarity of amino acid sequences for *Dm*HA1 and *Dm*HA2 was not high, the proteins are likely not isoforms of one protein and may have different functions. In this case, the *Dm*HA1 ATPase, which is similar to the well-characterized proton pumps of higher plants, is most likely a proton pump, while *Dm*HA2 could be a Na^+^-transporting ATPase.

Na^+^-transporting P-type ATPases have been identified functionally, as well as at the molecular level, in representatives of other eukaryotes: marine brown algae (kingdom *Chromista*) [69,70], protozoa (kingdom *Protista*) [71], green and red microalgae (kingdom *Plantae*) [41,72] and in primitive terrestrial plants, bryophytes [73]. Unlike the highly conservative H^+^-ATPase of P-type, the features of Na^+^-ATPases from various organisms may differ significantly. For example, the brown marine alga *Heterosigma akashiwo* and red alga *Porphyra yezoensis* have Na^+^-ATPases similar to Na^+^, K^+^-ATPases of animal cells [72,74]. Na^+^-ATPases in yeast form a special group of yeast-type ATPases (so-called ENA ATPases), which have low selectivity with respect to Na^+^ and K^+^ ions [47]. Na^+^-ATPases of protozoa are very similar to yeast ENA ATPases [71]. The Na^+^-transporting ATPase that was discovered in the green marine microalga *Tetraselmis viridis* [41], differs functionally from both animal Na^+^, K^+^-ATPase and yeast-type ENA ATPase. Unlike animal Na^+^, K^+^-ATPase that transport 3 Na^+^ per 2 K^+^, the ATPase from *T. viridis* does not transport K^+^ but uses the H^+^ ion as the counterion for Na^+^, i.e., during the catalytic cycle it exchanges Na^+^ for H^+^ with odd (not even) stoichiometry (the exact stoichiometry of the transport cycle is not yet known) [75]. In contrast with the yeast-type ENA ATPases with low selectivity between Na^+^ and K^+^ [47], the Na^+^-ATPase of *T. viridis* has high selectivity for Na^+^ [76].

**Figure 4:**
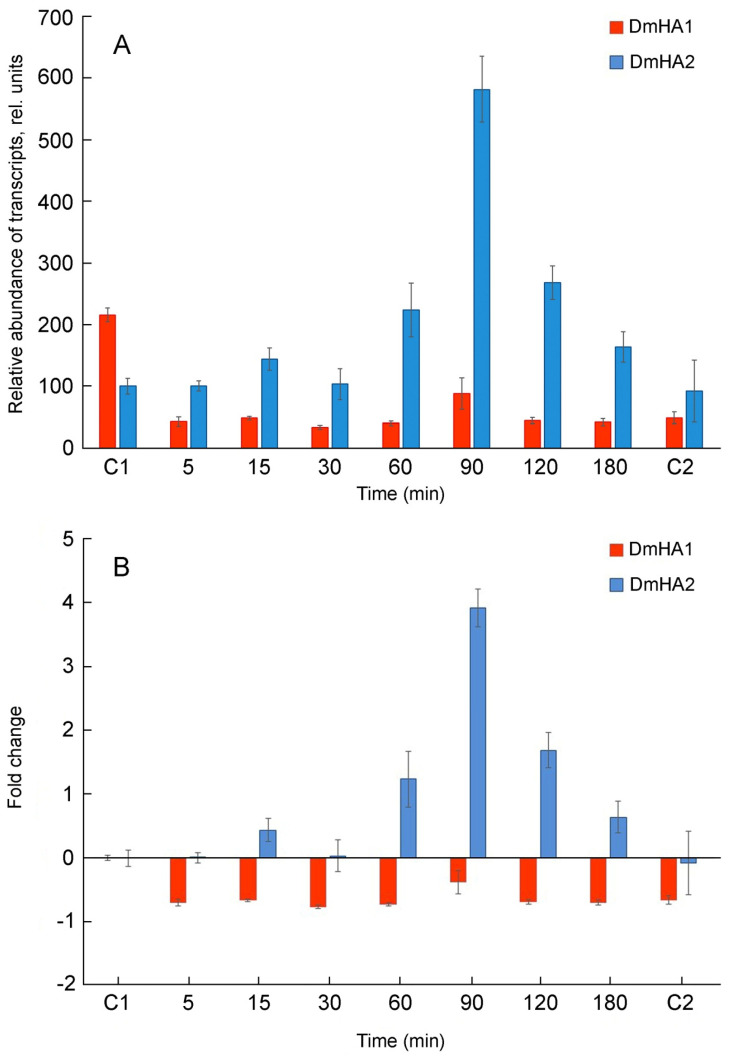
qRT-PCR analysis of *DmHA1* and *DmHA2* expression during the response of *D. maritima* cells to hyperosmotic salt shock. Sodium concentration in the medium was dramatically increased at “zero” time point. Points “C1” and “C2” represent the values of the expression of the ATPase genes in the algal cells growing at 0.1 M NaCl and 0.5 M NaCl in the medium, respectively. (**A**) relative abundance of transcripts; (**B**) recalculated fold changes of gene expression.

To identify a potential candidate for the role of Na^+^-transporting ATPase in *D. maritma*, the expression of the *DmHA1* and *DmHA2* genes under hyperosmotic salt shock was studied. Algal culture acclimated to relatively low salt concentrations (0.1 M NaCl) in the medium was used in these experiments. Hyperosmotic salt shock was created by adding NaCl to the cell suspension to a final concentration of 0.5 M. During the response of the cells to hyperosmotic salt shock, a significant induction of the *DmHA2* gene occurred, while the expression of the *DmHA1* gene decreased and mostly remained at a relatively low level (Figure 4): the level of *Dm*HA2 transcripts was significantly higher than that for *Dm*HA1 during the response of *Dunaliella* cells to hyperosmotic salt shock. The maximum expression of *DmHA2* gene was observed at 90 min after adding NaCl to the cell suspension. After 90 min, the level of *DmHA2* transcripts gradually decreased and returned approximately to initial values after 3 h. The expression of *DmHA1* slightly increased at 90 min (compared to 60 min) after the sharp rise of external Na^+^ concentration (Figure 4A). This increase of *DmHA1* expression could be explained by general biochemical reactions in response to hyperosmotic shock: they lead to synthesis of intracellular compatible osmolyte glycerol [9,77] and can acidify cytoplasm [78]. Plasma membrane H^+^-ATPase is a main player in the cytoplasmic pH-stat (e.g., [49]), hence its activation (including at the transcriptional level) is required to alleviate or prevent the cytoplasmic acidification.

**Figure 5:**
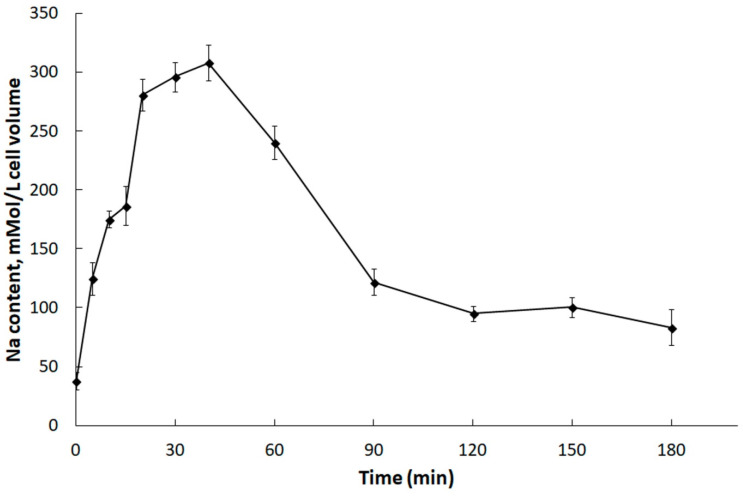
Changes in concentrations of Na^+^ ions in *D. maritima* cells under hyperosmotic salt shock. The alga was grown in a medium containing 0.1 M NaCl. At “zero” time, the salt concentration in the cell suspension was increased to 0.5 M. Each point on the graph is the average of 4 analytical replications. The abscissa axis indicates the incubation time of algal cells in hypertonic salt medium.

The dynamics of changes in the *DmHA2* gene expression corresponded to the dynamics of changes in intracellular Na^+^ concentrations in *D. maritima* under conditions of hyperosmotic salt shock (Figure 5). Immediately following the increase in the external salt concentration, an increase in intracellular Na^+^ concentrations (the so-called “entry phase”) was observed. After some time, the growth of intracellular concentrations of Na^+^ was replaced by a drop (“pumping phase”). Na^+^ concentration in *D. maritima* cells, increased as a result of the hyperosmotic salt shock, but returned to the new steady-state level by 90–120 min after the increase in salt concentration in the medium. This decrease of intracellular Na^+^ concentrations is accompanied by increased synthesis of glycerol [77] which finally becomes responsible for the osmotic balance in the algal cells [8,9,10]. The delayed maximum expression of *DmHA2* (at 90 min) compared to intracellular Na^+^ concentrations (peaked at 40 min) probably indicates a required threshold or specific signature of Na^+^ signal for induction of *DmHA2* gene while the excess Na^+^ ions are removed earlier by activation of pumping achieved mostly at translational or post-translational levels. The new steady-state level of intracellular Na^+^ concentration stabilizes though the expression of *DmHA2* decreases after 90 min of salt shock. It is likely that high expression of *DmHA2* is not necessary for cells under the new stationary conditions: Na^+^ entry might be limited now by low Na^+^ permeability of plasma membrane which is known for *Dunaliella* [7]. Therefore, it seems that intracellular concentration of Na^+^ under stationary conditions is rather regulated by membrane permeability than by high expression of ATPase. The observed kinetics of intracellular Na^+^ changes indicates that the mechanisms responsible for pumping Na^+^ ions out of alga cells work efficiently and are able to restore ionic homeostasis. A significant induction of *DmHA2* with an increase in the NaCl concentration in the medium indicates that the ATPase encoded by this gene is necessary for the alga cells to overcome hyperosmotic salt stress and, as a P-type transport ATPase, is probably directly involved in the export of Na^+^ ions from the alga cells, i.e., *Dm*HA2 might be a Na^+^-transporting enzyme. It is interesting to note (Figure 4A) that expression of *DmHA1* is higher than expression of *DmHA2* at 0.1 M NaCl in the external medium while the opposite pattern (higher expression of *DmHA2*) was seen for 0.5 M NaCl and that *D. maritima* grows less well at 0.1 M NaCl than at higher external 0.5 M NaCl (personal unpublished results). It is likely that under the low salt conditions (0.1 M external NaCl) the energization of the algal plasma membrane is insufficient for energy-dependent uptake of nutrients, which also often involves symport with Na^+^ ions [79,80,81], as the energization is realized by an electrogenic Na^+^-pump (e.g., [82]) (presumably *Dm*HA2). The low membrane energization could be a consequence of a low Na^+^ concentration gradient at the membrane. This low energization may require activation of the electrogenic proton pump (presumably *Dm*HA1), which also participates in generation of electric membrane potential; it then stimulates voltage-dependent nutrient transport processes. Higher external NaCl concentration (0.5 M NaCl), on the other hand, provides better opportunities for algal plasma membrane energization at the expenses of Na^+^-ATPase, so that the cells do not need the high expression of the H^+^-ATPase seen at the high concentration of NaCl in the medium.

## 4. Conclusions

The coexistence of proton pumps and Na^+^-transporting ATPases has been demonstrated for plasma membranes of many unicellular eukaryotes, for yeast, microalgal cells and protists [76,83,84,85]. The situation is likely typical for cells that live in media with rapidly changing ion (especially Na^+^) concentrations and pH; it demands the ability to activate different ion transport mechanisms to restore ion homeostasis of the cells.

Euryhaline green microalgae *D. maritima* inhabits shallow coastal basins and lagoons that are characterized by variable salinity [1,3]. Therefore, *D. maritima* is able to withstand sharp salinity changes and grow in a wide range of salinity [7,86]. Earlier, in experiments with plasma membrane vesicles isolated from *D. maritima* cells it was demonstrated using optical probes that both H^+^-ATPase and Na^+^-ATPase are functional in the *D. maritima* plasma membrane [51,59]. Both enzymes are P-type ATPases, which is typical for plasma membrane ATPases of eukaryotes. The data from the functional studies can be correlated with the results of the present research where coding sequences of two putative ATPases of *D. maritima*, *Dm*HA1 and *Dm*HA2, were cloned and analyzed. One of the enzymes, *Dm*HA1, is a conceivable proton pump since it shows high similarity to H^+^-ATPase of higher plants and to the H^+^-ATPase of *D. acidophila*. The latter is a freshwater species, its ATPase is overexpressed as the pH of the medium shifts to the region of acidic values, and the proton transfer function of the latter enzyme is beyond doubt [54]. Expression of *Dm*HA1 decreases and remains at a low level during the response of *D. maritima* cells to the hyperosmotic salt shock (Figure 4).

In contrast, expression of *DmHA2* increases under hyperosmotic salt shock. This indicates that the protein encoded by the gene is important for overcoming hyperosmotic salt stress experienced by the algal cells. The protein *Dm*HA2 is presumably a P-type ion transporting ATPase that takes a direct part in transport of Na^+^ ions; i.e., it represents a Na^+^-transporting ATPase. Results of our experiments investigating the expression of *DmHA1* and *DmHA2* agree well with the results of research where the changes in the plasma membrane proteome of *D. salina* were analyzed under hyperosmotic salt shock [87]. This proteomic analysis revealed upregulation of a protein similar to ATPase with GenBank ID ABB88698.1. A noteworthy observation is that *Dm*HA2 demonstrates high similarity with exactly this ATPase (Table 1).

Phylogenetic analysis showed that *Dm*HA2 is similar to the poorly-studied ATPases of other microalgae and parasitic protists, whose subclade on the phylogenetic tree is in the same clade with H^+^-ATPases of higher plants (Figure 2). However, the functions of the enzymes which are in the clade with *Dm*HA2, have not yet been experimentally characterized and their sequences are taken from the results on genome sequencing of the corresponding species. Hence, the characterization of the enzymes as H^+^-ATPases is still rather preliminary. The ion selectivity in three-dimensional protein structure is often determined by amino acids located on different transmembrane segments of the enzyme. They selectively coordinate the ion carried by the ATPase along the path of its movement through the membrane [88,89]. The ion-binding sites are unexpectedly similar for Ca^2+^-ATPases, Na^+^, K^+^-ATPase and H^+^, K^+^-ATPase; small changes in the ion binding sites of the enzymes are likely not the only reason determining the ion selectivity of these enzymes [90]. The binding of the “correct” ion neutralizes the negative charge in protein transmembrane segment and induces the conformation changes that lead to occlusion of the transferred ion [90]. Therefore, *in silico* prediction of ion selectivity for an ATPase requires experimental confirmation. For example, Na^+^-ATPase from yeast, ENA1, was initially identified as a putative calcium P-type ATPase on the basis of its sequence characteristics [91]. Two years later its capacity to extrude Na^+^, Li^+^ and K^+^ was reported [92]. Obviously, further research is needed to characterize functionally the cloned ATPases of *D. maritima*.

## Figures and Tables

**Table 1:** Molecular properties of *Dm*HA1 and *Dm*HA2 based on their deduced amino acid sequences (computed parameters).

Properties	*Dm*HA1	*Dm*HA2
Protein accession number (GenBank ID)	QEH60479.1	AQM50087.1
Number of amino acids	1131	923
Subunit size (kDa)	123.4	100
pI	5.35	5.62
GRAVY index *	0.030	0.166
Subcellular localization	plasma membrane	plasma membrane
The proteins with highest homology scores **	P54211.1 (100% identity) *** P54210.1 (75% identity) ***	ABB88698.1 (99% identity) ***

* GRAVY (Grand average of hydropathicity) indicates the solubility of the proteins: positive GRAVY is for hydrophobic proteins; negative GRAVY is for hydrophilic ones. ** Identity for aa sequences is shown. *** P54211.1, H^+^-ATPase *D. bioculata*; P54210.1, H^+^-ATPase *D. acidophila;* ABB88698.1, P-type ATPase *D. salina.*

## Data Availability

Available data are presented in the manuscript and supplementary material; the sequences are deposited in GenBank.

## Supplementary Materials

The following are available online at https://www.mdpi.com/article/10.3390/plants10122667/s1, Table S1: Primers used in the study, S2: Contigs from the *de novo* assembled transcriptome of *D. tertiolecta* containing the coding sequences for *Dt*HA1 and *Dt*HA2 ATPases, S3: Partial coding sequence for *Dm*HA2, S4: Alignment of aa sequences of DmHA1 (QEH60479.1) and DmHA2 (AQM50087.1) ATPases from *D. maritima*. The identical amino acids are shown in black, and the conserved amino acids in P-type ATPases are highlighted by frames.
