# Supplementary material for: Cloning and Characterization of Two Putative P-Type ATPases from the Marine Microalga Dunaliella maritima Similar to Plant H+-ATPases and Their Gene Expression Analysis under Conditions of Hyperosmotic Salt Shock
Source: Plants (Basel). 2021 Dec 3;10(12):2667. doi: 10.3390/plants10122667 (PMC8708325; doi:10.3390/plants10122667)

Supplementary materials

Table S1. Primers used in the study.

Primer	Primer sequence (5' – 3')
Primers used for cloning the full-length CDSs of the ATPases *)	
DmHA1_ F1	*ataaacaaaccatgg*TTCCGCACCTGTTCACGAG
DmHA1_ R1	*acccgtagggcttga*ACCAAAGGTTTCACATGCACT
DmHA2_ F1	*acacacataaacaaacc*ATGGGCCTGACCATTGAGCCC
DmHA2_ R1	*acccgtagggcttga*TCAGGCCTGCTTCTCACCC
Primers used in qRT-PCR analysis of ATPase expression	
DmHA1_F2	GCGCCCTGTCTGCCAACACA
DmHA1_R2	TCAGCACGCGGAACACCCT
DmHA2_ F2	AGGATGATGTGCTGCTTTACTCC
DmHA2_ R2	GTGATCAGCTTCTCGCCAGT
β-tub _F	CAGATGTGGGATGCCAAGAACAT
β-tub_R	GTTCAGCATCTGCTCATCCACCT
Primers used for linearization of pMB1 vector	
pMB1_R	CATGGTTTGTTTATGTGTGTTTATTCG
pMB1_F	TCAAGCCCTACGGGTCATGGGCCT

*) Primers at the 5' end carry 15-17 nucleotides for cloning into the linearized vector pMB1.

S2. Contigs from the *de novo* assembled transcriptome of *D. tertiolecta* containing the coding sequences for *Dt*HA1 and *Dt*HA2 ATPases

Original sequence: (for *Dt*HA1)

```
- 4971 nucleotides
tgaggggtgaataaatagatgtttcttgcttggtgtgtgtgaacaaataattacaatttg
gcagtcacctctgtgggcgcacacggagtggtgggcttgcttcctggctccagagtagtt
ccgcacctgttcacgagcaaatggcgcacctgtgaggcccgctgcgtgcaagtgcgcctt
cttcgcgccacactcccacctttgacacgcagacacagagcgccctgacctgtcagagct
tgctttaggccgtgagctgccaaccgactcagccgtctctgcctggagagggcccctggg
cccgctgagtgccgcaacatggcggacatcaaggaaggagtcgaggaaggatcggtgaag
gtggacatgatcaaggagcccctcacacaaggggacactggcgtggatgaggtggacttt
gccaagattactctggacgatgccttcaagtacttgaattgcaacaagcacgggctcagc
agtgccgaagcagctgctcgtcttcaacagcacgggcccaacaagcttcctgacagttca
cgcaaccctgtccttgtcttccttggatacatgtggaacccctggcgtgggccatggag
gcagccgcaatcatctccattgccctcctggatgtggcagatttcgtgctcattgtgggc
ttgctgctcatcaatgccattatcagtttctatgaggagagcaacgccgacaaggccatc
aaggccttgacagctgcccttgcacccaaggccatggtcgtgcgagatggtgccattgtg
accatcgatgctgtgaatctcgtgccggggacgtcatcttaatccgcttgggcaacatc
gtaccagcagacgtcaagctgctggaagaagagggagctgatgagggggagcaggaagcg
cccatgcagatcgaccaagccgccctcacaggagagtcccttccggccaagaagttcacg
ggcgacgtggccttcagcggctcgagcatcaagcagggagagcgccatgcagtggtgtat
gctactggtgtgaacaccttcttcggacgtgcagctgccctcatcagcggcaccaacaac
gtatccaacctgcagactgtcatgaacaagatgagcgccatctgcatcgtcaccatcctg
ctgtgggtcgttgtcgagctggccgtgcaatttgggcactactcgcatgaatgcgttggt
ggcagagagggctgccccaccctgctgaatatgctggtggtgctggtgggcggtattccc
attgccatgcccactgtgctgtccgtgaccctcgccctgggtgcctacaagcttgcacgc
gagggtgccatcgtcacccgtatgagcgccgtagaggagatggcaggcatggatgtgctg
tgctctgacaaaaccggcaccctcaccttgaacaagctgtccattgacaagagcatggtg
gtgccggtgggcaacatgggcgtggatgagatcatgagaatgggcgccctgtctgccaac
acagtcacagaggagcccatcgatatggtgctgtgggagtcttatccagacagggaaaca
attaagagggactacaagcacaccaagtacttccccttcaaccccaatgacaagattacc
atcgcaacgtgcctggagatcgccaccggcagggtgttccgcgtgctgaagggctctcct
caggtggtgctggccaaggcgtggaatgcagccgagctggatgccaccgtgaaccagaag
atggtggaatttgcaaaccgcggcttccgcgcgctgggcttggctatggcagacggcgac
ggcaaagatggcaccaagtgggagatgctggcgctgctgccgctgtttgaccccctcgc
cacgacaccaaggagaccatcgagcactgccagaaccagggcatccaagtcaagatgatc
actggtgaccacttgcttatcgggaaggaaaccgccaagatgctgggcatgggcactgag
atgttccccagtgaggtcatgatcaaggcccgcaatggcgacgcaagccagctgcacggc
tacaagaactttgtggagatggtggagacctgcaacggctttgcccaggtgttcccggag
cacaagtttgagatcgtcaagatcctgcaggactccaaccacgtcgtcggcatgacaggt
gatggtgtgaatgacgcacccgccctgaagaaggctgacgtgggtgtggctgtggctgac
gccaccgatgctgctcgtggtgctgccgacatcgtgctgacggagcctggcttgtccacc
atcgtgacggcggtgatcggcgcgcgcaagatcttccagcgcatgaccacctactccaag
tacaccatcgccatgaccttccgtatctgcttcacctttggcctgatcaccgtcatctac
gactggtacttccccaccatcctcatcgtcatcatggctgtcttcaacgatggtgccatg
attgcgctgtctaaggaccgtgtggtggcctccaagacgcccaatagctggaacatcacc
aacatcttcatcatgggcatggtgtacggcctgtacctcaccctctccacatgggccttg
taccagactgccaccaagaccacgttcttcgaggacaagacacccttgcattcactcaat
gaccagtacagcgtcctgcagccctggtgtgaggacgaagtgcgggccaagcttggacaa
accatcgacccctacgcctcactgtgcgagtccaacagctacgccaagcagtttgacgag
tgcgagggataccagaagggctcaggcgtgcaggtggaggacgtccctaccctgcatgcc
caatgcgtgactgagcaacgttacctgcgtggcgccatgacgcgctccctcatctacacc
caggtctcaatttctggtcaggccctcgtgtttgtcgtccgtactgcgggctactccttg
atggagcgcgcgggcacctccacatacctggccttcttctttgcccaggtgggcgccacg
ctgtttggtatctttggcctgggtggctttgagaagccccgccaccagctggaggactgc
cagttctgcgactactccttccatgagcccgtagactggtttgactccgggattgtgcct
gagtccggcacagagtccgacttcactgcctctgtcatcggatgcggtggttacgtgatt
gtggcctggatctggtctgccatttggtacgtgctgctggaccccatcaagtggatcctg
```

```
ttctggatcttgaacgaggagggcttcagggacacgatgtcctggcgcgagagcaccaag
aggagcctggaccgccgcagcaaggatgacatcggcgacaaggagttcacggggccctct
ggcatggtgccggccaactactccaacccсctgggccgtgcgtccatgtccaagcctgtg
tcagctgtgctggaccgcaagtccgcctccctggttgctatcaaccgcaactctatgact
gtgagccaggaccccaaccgcgcgctcaacatcggccggcgctccatgattggccgcccc
tctgggcctgttggccgcacgtccatgcccttgggccgtatctcgcgcacttccaacacc
ttgtccacaggctctaaggatggccagatcggcagaggaagcaagcctctgaacagctcg
tccgctgagatcaagcccgacaagtatgacttcgcttccaccatcagggagtgagccctc
tctctctcgtcagcaagctgtcaaaagctgttgagagtgattgggtgacccccatgaatg
gataatggagagtgcatgtgaaacctttggttccagcaagcaggggcagaacacccttgc
cttagttcgaacaaactggccatcagggttgatcctcctgtgcaggtagagggcgcgtcc
ttcagcgctccactccttgtctgagtgcacatacgtgcgtgcctgcacctgttctcaccc
attctccctcattgttgcaatgtgaaagtattgctgtgctcatatgatgattcactttc
aacagctagggagcagctaggcgcaatgtgtgcgctcttttagcaaggctgctaggtcag
tgaacaacaactagggagcagcttggggcgctaagtgcgcactgatgggcaaagctgctc
aatcagtgaacgtatgctagagaatttaaatacccgcagttcgcaggattatgcacctct
ccgtgtgtcctgcaagtgtgggcatgcgcagcactttgcagggatatgctcttctgtgtg
tgttttgcaagtgtgggcttgcgcacccatgctccacagatatccgtgtgtggcgaagag
gttggtggacatgctttcatagttgcgacttgtttcatgcttttgaaggggagcaagcca
aagttttagcaatgctgctgcatatgtctctggattgtgtagaggctaccttattctgcc
atccgcttcctctagagcatgtgcttacctcttgtgtaaggagttcttgctactgattac
ctcggtgaggctttgctcgcgtgtgctcgcatgctcacaggtgcggctattacgcaatgt
tgcctgcattttgcttctttatcccaattcctttgtgtcttctctggtgcttttctgtga
cgcaagctttccaccttaattgttcttgcggctcaacacaactgcatgcatgagtgagac
atgcatccagaaagacgcagaacccaggaatttgtgttaggataacttacttatgaagcc
tttaccggtattaatacaggtattaggtaaatcagcgtgatcagcttgtggaacacgtat
gactattatgtgttgcatcgttctgatgagtattttcatcattgatgcacccttttggt
attcattgctgggatttgcgtgatttggtattcattattccttgtaacagagtgagcata
atgcaattccaggtcttcttcacaggcgagttttaacttcttgatccgcag
```

Web.expasy.org/translate/

```
SEQUENCE   1131 AA;  E493726BD5CD732D CRC64.
     MADIKEGVEE GSVKVDMIKE PLTQGDTGVD EVDFAKITLD DAFKYLNCNK HGLSSAEAAA
     RLQQHGPNKL PDSSRNPVLV FLGYMWNPLA WAMEAAAIIS IALLDVADFV LIVGLLLINA
     IISFYEESNA DKAIKALTAA LAPKAMVVRD GAIVTIDAVN LVPGDVILIR LGNIVPADVK
     LLEEEGADEG EQEAPMQIDQ AALTGESLPA KKFTGDVAFS GSSIKQGERH AVVYATGVNT
     FFGRAAALIS GTNNVSNLQT VMNKMSAICI VTILLWVVVE LAVQFGHYSH ECVGGREGCP
     TLLNMLVVLV GGIPIAMPTV LSVTLALGAY KLAREGAIVT RMSAVEEMAG MDVLCSDKTG
     TLTLNKLSID KSMVVPVGNM GVDEIMRMGA LSANTVTEEP IDMVLWESYP DRETIKRDYK
     HTKYFPFNPN DKITIATCLE IATGRVFRVL KGSPQVVLAK AWNAAELDAT VNQKMVEFAN
     RGFRALGLAM ADGDGKDGTK WEMLALLPLF DPPRHDTKET IEHCQNQGIQ VKMITGDHLL
     IGKETAKMLG MGTEMFPSEV MIKARNGDAS QLHGYKNFVE MVETCNGFAQ VFPEHKFEIV
     KILQDSNHVV GMTGDGVNDA PALKKADVGV AVADATDAAR GAADIVLTEP GLSTIVTAVI
     GARKIFQRMT TYSKYTIAMT FRICFTFGLI TVIYDWYFPT ILIVIMAVFN DGAMIALSKD
     RVVASKTPNS WNITNIFIMG MVYGLYLTLS TWALYQTATK TTFFEDKTPL HSLNDQYSVL
     QPWCEDEVRA KLGQTIDPYA SLCESNSYAK QFDECEGYQK GSGVQVEDVP TLHAQCVTEQ
     RYLRGAMTRS LIYTQVSISG QALVFVVRTA GYSLMERAGT STYLAFFFAQ VGATLFGIFG
     LGGFEKPRHQ LEDCQFCDYS FHEPVDWFDS GIVPESGTES DFTASVIGCG GYVIVAWIWS
     AIWYVLLDPI KWILFWILNE EGFRDTMSWR ESTKRSLDRR SKDDIGDKEF TGPSGMVPAN
     YSNPLGRASM SKPVSAVLDR KSASLVAINR NSMTVSQDPN RALNIGRRSM IGRPSGPVGR
     TSMPLGRISR TSNTLSTGSK DGQIGRGSKP LNSSSAEIKP DKYDFASTIR E
```

Original sequence: (for *Dt*HA2)

```
> - 4150 nucleotides
ttgagctaggagtcactaggattattggccgggcgaacacaggaaagacaggcagcagca
gcatctgcggacactcactgcctttttgcagcccagacctgcacgaacgacaagggagac
agccccgccagagcagcaggctcaaatgggcctgaccattgagcccccccatgatcacgg
actgacaacccaggaggttgagcagctgcagaaggagtggggtctcaaccatgtcgctgc
caagacgatcccggagtggaagaaaatccttgatcgctacctggactgggtgtcgctcat
cattctcatttccgccatcatttccgcggcggtgcccgtcaatggcgaccagggctggac
ctcctttgtgatgctcatcctcgagctgcagttcgtggtgtggatgggctactactcgga
ccggaatgcgggagatgccgtcgctgagcttgcggccctgtctgcacccatgtgccactg
cttgcggaacgggaagtggggcagcctgccggtgaaggagctggtgcctggcgacatcat
tggcctcaagggtggtgatgtcatcccagcagacagtaagctcattggagagggagagcc
cctgaagattgatgagtcctcactgacaggggagtgccttgcagtcacgaggcaccctgg
ccaagagattcttgcgggtgctgtggtggtgtctggtgagctggacgccatggtcactgc
tactggtgtgaactccttctttggtaagacaatggccttgctggccgtcccccccgagcg
tggacacttgcagcaggtgctcaaccgtgtgtccattgccctggccctgtttgccgtcgc
tggctgcgccatcattctgggtgtgctcaccggtcactacgacaaccccccctgggtattc
catcgtcactgtcttcgtcattttcacctccgtggtgcccattggcatgcctgtggtcac
caccactgtgctggctgtgggtgcccgggagatggcccgcgagaaggccattgtcacaag
gctgtcagccctggaagagatgtctggtatggaggtgctggcttccgataagactggcac
tctcacccttaaccagctgagccttgacaaggaggacatcctgaactggggtacccatac
caaggatgatgtgctgctttactcctgcctgagtgccaaatgggagaacaatgatgccat
cgataaagccgtgaccaactccctgggagacaagaagtatgttgccggctacaagatcac
caagttcagccccttcaaccccgtggacaagaagaccaccgcccacaccatcacccccac
tggcgagaagctgatcaccaccaagggtgcccccagatcattggtgacatgctggctga
ccctgctgcacgccaggcctgcgcagactacattgcagagcgcgcctcccgcggcctgcg
ctccctgggtgttgcccgctccgatgatgatggccagacctggtccctggtgggcctcat
ctccttgctggacccccctcgccctgactctggagagaccatcaagctggcccagtccat
gggtgtggcagtgaagatggtgacaggcgaccagtttgccattgccgtggagacctgcaa
gcgcctgggcatgggctctaccatcatggagggcaagacggtcatggcaggcctgaaggg
cggcgatgagggcaagcctgaccctgtcctgatccagcactgcgacgagagtgatggctt
tgccggcgtgtacccggagcacaagcacatgattgtgtcagcattgcaggccaagggggcg
cctggttggtatgacaggtgatggtgtgaacgatgctcccgccctgaagaaggccaacgt
cggtattgccgtcgctggtgccacatctgctgccaagggtgctgcagatatcatcctgac
cagggagggtatcagcaccatcatcattgccatcgtgcgctcccgcaagatcttccgccg
cctggagatgtacatcatctaccgcatggcctcctccgtgctcatcctgggcttcttctt
ctttgccatcctcatctttgacttcgagatccccacctggatcctggtgctgatttccat
gctcaacgacgcctccgtcattgccacctcctacgacgcggtgcacagctctgactaccc
cctgcactggaacatgaccaaggatctggcgattgccttctccattgccatggtgggcat
tgtgggcaacgtgctgctggtgcccttcgtccgccctgatctgtggtttgagtggcctga
gcttgacaccgagcctgcgctcaagacccccccctgacaatggcgtgtccacctctggcaa
ggagtcggccctgatcttcctgtccctctccggcatggtccagctgaacatcatcctgac
ccgcaacccctccttctggtggcacttcagcaagaagagcgcccccaagccgtcccccat
cttgctggtccctgtgacatgcttcctgggtggttccaccttcatgtccgtgtactggaa
cggcaacatcaagcctgacggacagcgctacctgtttgagggcgcaggctggcacgcggt
gctgctggtgtgggcctatgttttcgtcttctgggtcatcgctgacttcttcaaggtggc
catcagctccgtcttcgtgaaggccgacctgatcaaggatgagctcaagggccacattga
tggtaaggagaagacccccggctgggtcaaggccctggactggcctggtgagaccgccga
caagatcagtgacaagattgaggcctgcttcgacggcatgtgctcttgctttgagaagaa
agagaagaaggccaagttccagcgcacgtccgtcgtgtctgagaaggagggagagggcca
ggtgcatgtgcaagtggagggtgagaagcaggcctaatcaagccctacgggtcatgggcc
tgctcatgcagtgagcgcatgctcagctgcaggtacgtcacgcagcatgagccacaatcc
gaatgtctgctcgtgtggtttggaaggggaaggcaggcgcacgatggagggggggttgcca
gcttacattttgtctcgagtgcgcctcttttgcttggttgtagagtgacttggtggttg
aggcagacggagggcctcccatcgatcaatcccacacgattctcttttactgtgcgttaa
agttttgccaaactaggccgtacacacctgcccgctcacgccattttgtttggcttcctg
gcaatgagttgtagcctgtgagcgcatttaaaagtactgcatatgcactgttgtgctata
ggatctaaaacaacaatggaggcgtgcgtgtctgttgtttgtcgtggaggcacgtgctca
tgcatgcaattgtcctggtgcctcaggcctcctcccctccttgtatgaaagattgctttt
tagcttagcgtctgatttatggcggtggaggaggagacaacatagcagcagagtttctac
ggtgttgtggcgtgtgctcatccgttagcattctacagttgccacgctaggttgctgcct
```

```
ccgggcatgttccagttggtcctctcagattgcatgatgcaatttccgtctgaaagcatg
cgtgagggcaccttctgccctggcatacatacatgtagctgtcttccacagtggatgggt
aggtagttccggaatgggcgggcacgcgccatgaccagctgggtccactagggtcaggag
atgcccagtcccgctatccctccatcttgcgacgcatgaaacacatcctgagtgtcggct
ttgcaggagtgtgttccaggagcaagctccagcttgctttatgaggcaggggaatgggtg
tcaatgcccagcgcgtgcccttcctgaaggcaggggctggccggcatgcctatagtatgt
actttaatttccgtatgatttgttcagcaggctgtgtgtttgctcgataaactctcattt
tattgcggtcatctggacctgtttctacactcaaaacctaactttcaactctatagggtg
ttggcttttacacttcggttgtgcacgagaaaggtagcctggttttcagtggggcgctgt
aagtgtacacagtttcctgcaatccctagtccagcagcttcttcggtttctccaatttca
gtccataatg
```

Web.expasy.org/translate/

```
SEQUENCE   923 AA;  098AE9E1B61820DA CRC64.
     MGLTIEPPHD HGLTTQEVEQ LQKEWGLNHV AAKTIPEWKK ILDRYLDWVS LIILISAIIS
     AAVPVNGDQG WTSFVMLILE LQFVVWMGYY SDRNAGDAVA ELAALSAPMC HCLRNGKWGS
     LPVKELVPGD IIGLKGGDVI PADSKLIGEG EPLKIDESSL TGECLAVTRH PGQEILAGAV
     VVSGELDAMV TATGVNSFFG KTMALLAVPP ERGHLQQVLN RVSIALALFA VAGCAIILGV
     LTGHYDNPPG YSIVTVFVIF TSVVPIGMPV VTTTVLAVGA REMAREKAIV TRLSALEEMS
     GMEVLASDKT GTLTLNQLSL DKEDILNWGT HTKDDVLLYS CLSAKWENND AIDKAVTNSL
     GDKKYVAGYK ITKFSPFNPV DKKTTAHTIT PTGEKLITTK GAPQIIGDML ADPAARQACA
     DYIAERASRG LRSLGVARSD DDGQTWSLVG LISLLDPPRP DSGETIKLAQ SMGVAVKMVT
     GDQFAIAVET CKRLGMGSTI MEGKTVMAGL KGGDEGKPDP VLIQHCDESD GFAGVYPEHK
     HMIVSALQAK GRLVGMTGDG VNDAPALKKA NVGIAVAGAT SAAKGAADII LTREGISTII
     IAIVRSRKIF RRLEMYIIYR MASSVLILGF FFFAILIFDF EIPTWILVLI SMLNDASVIA
     TSYDAVHSSD YPLHWNMTKD LAIAFSIAMV GIVGNVLLVP FVRPDLWFEW PELDTEPALK
     TPPDNGVSTS GKESALIFLS LSGMVQLNII LTRNPSFWWH FSKKSAPKPS PILLVPVTCF
     LGGSTFMSVY WNGNIKPDGQ RYLFEGAGWH AVLLVWAYVF VFWVIADFFK VAISSVFVKA
     DLIKDELKGH IDGKEKTPGW VKALDWPGET ADKISDKIEA CFDGMCSCFE KKEKKAKFQR
     TSVVSEKEGE GQVHVQVEGE KQA
```

S3. Partial coding sequence for *Dm*HA2.

```
  1 tgttcggaca agacggggac tctgaccctg aacaaattgt ccattgacaa gagcatggtc
 61 gtgccagtgg gccccatggg cgtggacgaa atcatgaaga tgggcgcatt gtctgcgaac
121 acggtcacgg aagagcccat tgacatggtg ctgtgggagt cctacccaga gagggatacc
181 atcaagaggg actacaagca taccaagtac ttccccttca accccaacga caagatcacc
241 attgcgacgt gcctggaata cgctactggc aaggtcttcc gggtgctgaa gggttctcct
301 caggtggtgc ttggcaaggc gtggaacgca gcagagctgg atgccactgt gaaccagaag
361 atggtggact tcgccaaccg cggcttccgt gcgctgggcc ttgccatcgc tgacggagat
421 ggcaaggagg gaacaaagtg ggagatgctg gcgctgctgc cactgtttga ccctccccgt
481 cacgacacaa aggatactat tgagcactgc cagacacagg gcatccaagt caagatgatc
541 acgggcgacc atttgctaat tggaaaggag actaccagga tgctgggcat gggcactgag
601 atgttcccca gtgaggtcat gatcaaggcc cgcaacggtg acgcgagtca gctgcacggc
661 tacaagaact ttgtggagat ggtagagacc tgcaacacgt tcgcccaggt gttccccgag
721 cacaagttcg agattgtcaa gatcctgcag gacgccaacc acgttgttgg catgaccggc
781 gacggcgtca acgat
```

S4. Alignment of aa sequences of *Dm*HA1 (QEH60479.1) and *Dm*HA2 (AQM50087.1) ATPases from *D. maritima*. The identical amino acids are shown in black, and the conserved amino acids in P-type ATPases are highlighted by frames.

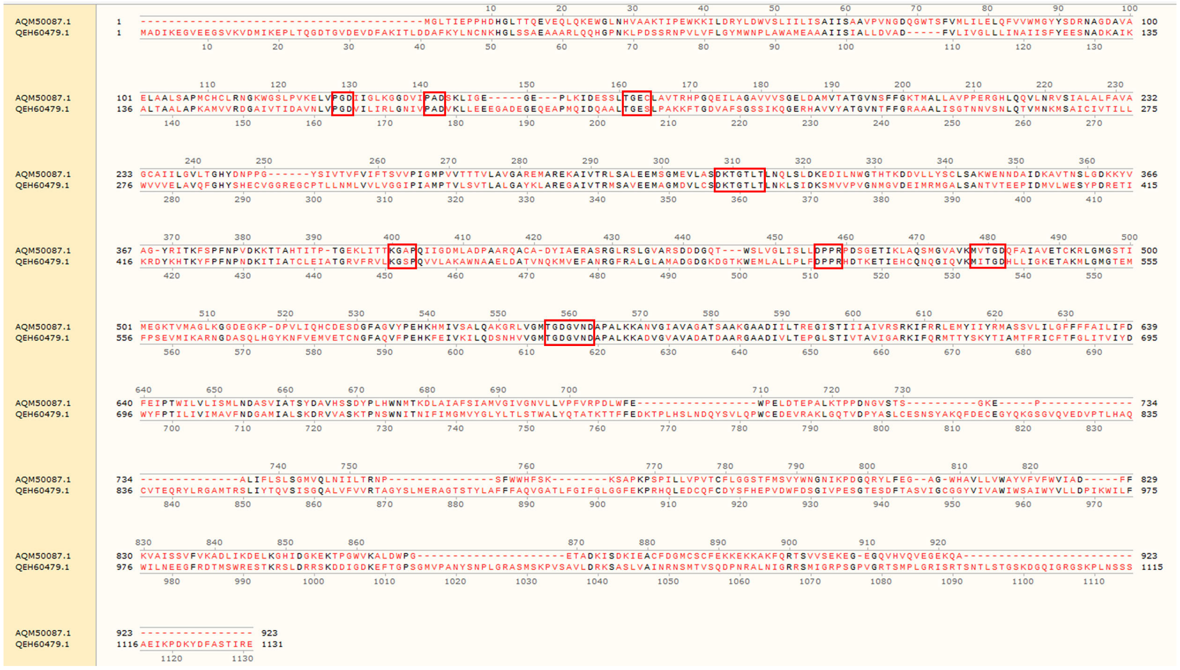

Supplement: Supplementary file 1 [file plants-10-02667-s001.zip › plants-1462028-supplementary.pdf]
